# Genetic Landscapes Reveal How Human Genetic Diversity Aligns with Geography

**DOI:** 10.1093/molbev/msz280

**Published:** 2019-11-28

**Authors:** Benjamin M Peter, Desislava Petkova, John Novembre

**Affiliations:** 1 Department of Human Genetics, University of Chicago, Chicago, IL; 2 Max Planck Institute for Evolutionary Anthropology, Leipzig, Germany; 3 Wellcome Trust Center for Human Genetics, University of Oxford, Oxford, United Kingdom; 4 Department of Ecology & Evolution, University of Chicago, Chicago, IL

**Keywords:** population structure, population genetics, human genetics, isolation-by-distance, geography, geographic structure

## Abstract

Geographic patterns in human genetic diversity carry footprints of population history and provide insights for genetic medicine and its application across human populations. Summarizing and visually representing these patterns of diversity has been a persistent goal for human geneticists, and has revealed that genetic differentiation is frequently correlated with geographic distance. However, most analytical methods to represent population structure do not incorporate geography directly, and it must be considered post hoc alongside a visual summary of the genetic structure. Here, we estimate “effective migration” surfaces to visualize how human genetic diversity is geographically structured. The results reveal local patterns of differentiation in detail and emphasize that while genetic similarity generally decays with geographic distance, the relationship is often subtly distorted. Overall, the visualizations provide a new perspective on genetics and geography in humans and insight to the geographic distribution of human genetic variation.

In many regions of the world, human genetic diversity “mirrors” geography in the sense that genetic differentiation increases with geographic distance (“isolation by distance” Ramachandran et al. 2005; Novembre et al. 2008; Wang et al. 2012; Bradburd and Ralph 2019; Battey et al. 2019); However, due to the complexities of geography and history, this relationship varies across the globe. Pioneering studies of classical blood group and allozyme loci (Barbujani and Sokal 1990; Cavalli-Sforza et al. 1994), mostly across Europe, found that some allele frequencies exhibit zones of elevated change that frequently align with each other. Later studies of large microsatellite marker panels (Rosenberg et al. 2002) observed broad geographic clustering, which lead to a debate whether human fine-scale genetic variation is better characterized by discrete clusters or continuous clines (Serre and Pääbo 2004; Rosenberg et al. 2005; Frantz et al. 2009; Perez et al. 2018). Since those early studies, methods in spatial or landscape genetics have matured, with new, powerful methods capable of modeling population structure allowing for spatial heterogeneity (Guillot et al. 2009; Bradburd et al. 2016; Novembre and Peter 2016; Ringbauer et al. 2017; Bradburd et al. 2018; House and Hahn 2018; Ringbauer et al. 2018).

One of these methods is the tool EEMS (for Estimated Effective Migration Surfaces, Petkova et al. 2016). EEMS uses a model based on local “effective migration” and “diversity” parameters. Importantly, it is a model-based visualization tool. The parameters of the model are not intended to be interpreted literally—they are simply tools to help visualize the relationship of genes to geography. Populations in areas of high effective migration are genetically more similar than other populations at the same geographic distance, and conversely, low effective migration rates imply genetic differentiation increases rapidly with distance. In turn, a map of inferred patterns of effective migration can provide a useful visualization of spatial genetic structure for large, complex samples.

To date, the EEMS method has not been applied to human diversity data from very large, spatially extended samples. The method has the potential to produce useful summaries of human genetic variation that are more transparent and immediately interpretable than alternatives using methods such as principal components analysis. To explore this possibility, we have applied EEMS and PCA using single-nucleotide polymorphism (SNP) data combined from 27 different data sets comprising a total of 6,066 individuals from 419 locations across Eurasia and Africa (supplementary information, Supplementary Material online).

We organize our applications in seven analysis panels: an overview Afro-Eurasian panel (AEA), four continental-scale panels, and two panels of Southern African KhoeSan and Bantu speakers. In all cases, the inferred EEMS surfaces are “rugged,” with numerous high and low effective migration features (figs. 1*a* and 2) that are strongly statistically supported when compared with a uniform-migration model (supplementary table 2, Supplementary Material online). The regions of depressed effective migration often align in long, connected stretches that are present in >95% of MCMC iterations. We refer to these features as “troughs” and annotate them with dashed lines (figs. 1*a* and 2, supplementary figs. 2*a* and 3, Supplementary Material online show these troughs in isolation, supplementary figs. 2*b* and 4, Supplementary Material online show the posterior variance on migration rates).

**FIg. 1:**
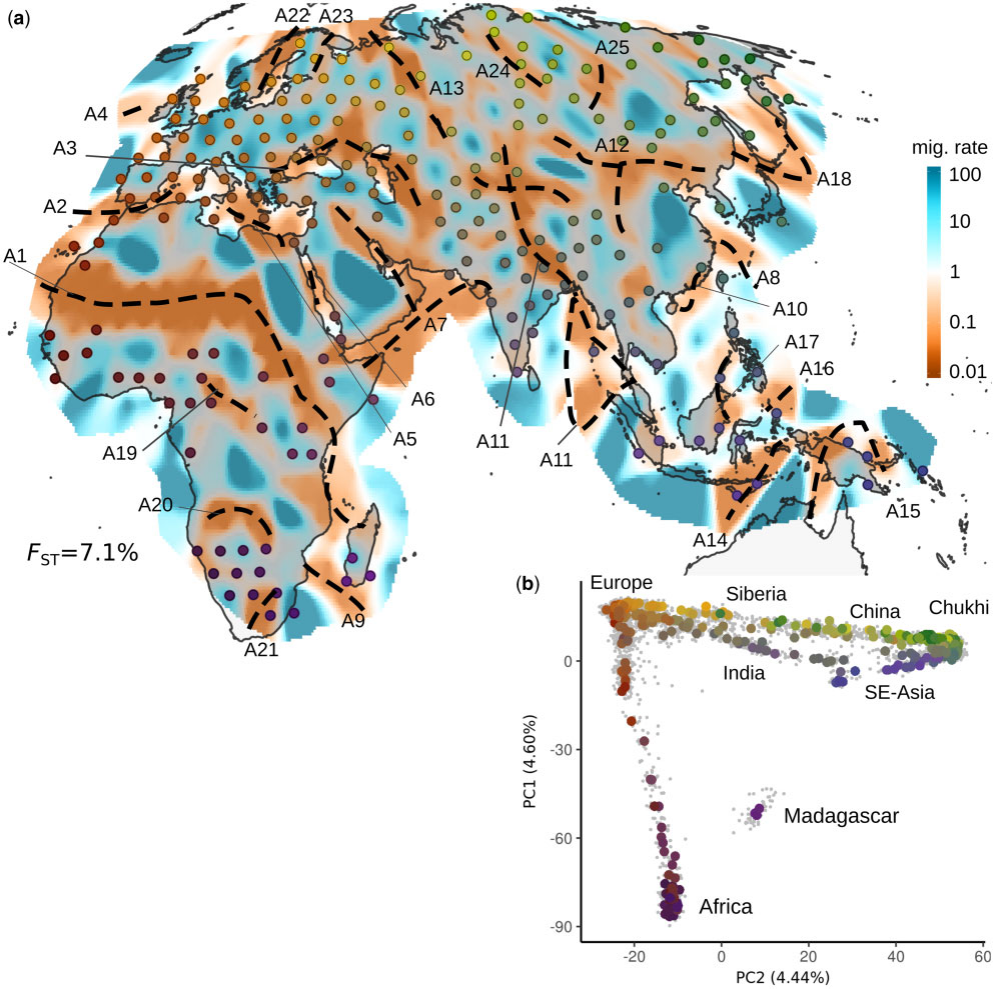
Large-scale patterns of population structure. (*a*) EEMS posterior mean effective migration surface for Afro-Eurasia (AEA) panel. Regions and features discussed in the main text are labeled. Approximate location of troughs is annotated with dashed lines (see supplementary fig. 2, Supplementary Material online). (*b*) PCA plot of AEA panel: Individuals are displayed as gray dots, colored dots reflect median of sample locations; with colors reflecting geography and matching with the EEMS plot. Locations displayed in the EEMS plot reflect the position of populations after alignment to grid vertices used in the model (see Materials and Methods). For exact locations, see annotated supplementary figure 2, Supplementary Material online and supplementary table S1, Supplementary Material online. The displayed value of *F*_ST_ emphasizes the low absolute level of differentiation in human SNP data.

**FIg. 2:**
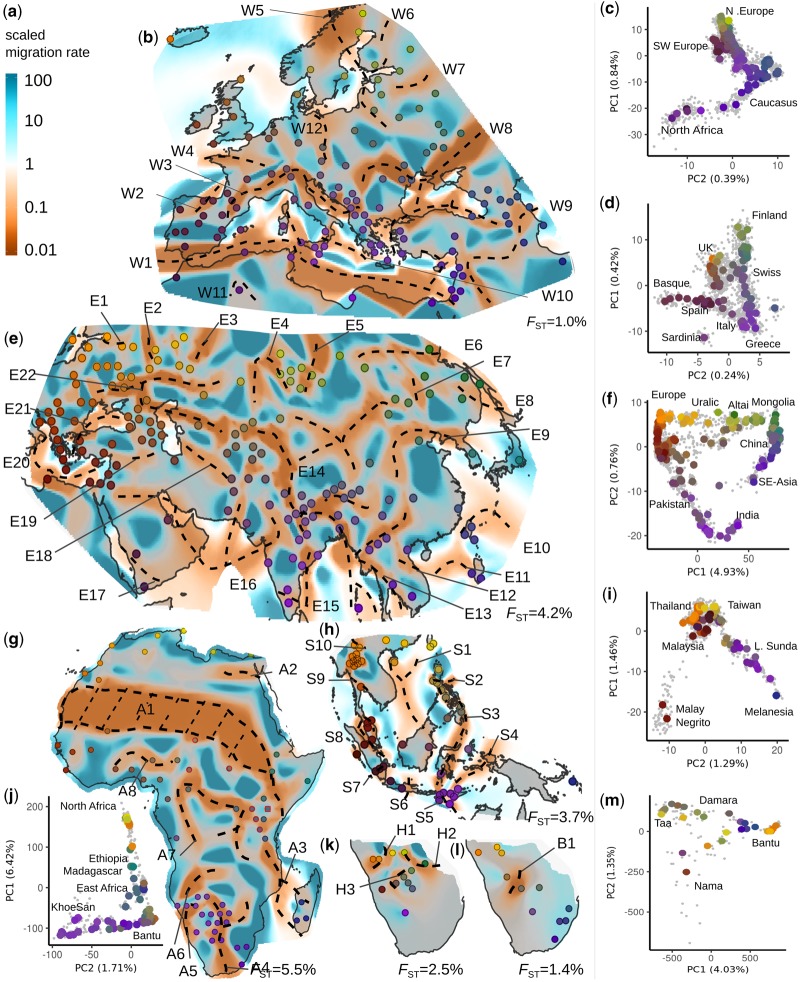
Regional patterns of genetic diversity. (*a*) Scale bar for relative effective migration rate. Posterior effective migration surfaces for (*b*) Western Eurasia (WEA) (*e*) Central/Eastern Eurasia (CEA) (*g*) Africa (AFR) (*h*) South East Asian (SEA) (*k*) Southern African KhoeSan (SAKS) (*l*) Southern African Bantu (SAB) analysis panels. In panel *g*, red circles indicate Nilo-Saharan speakers. Approximate location of troughs is shown with dashed lines (see supplementary fig. 4, Supplementary Material online). PCA plots: (*c*) WEA (*d*) Europeans in WEA (*f*) CEA (*i*) SEA (*j*) AFR (*m*) SAHG+SAB. Individuals are displayed as gray dots. Large dots reflect median PC position for a sample; with colors reflecting geography matched to the corresponding EEMS figure. In the EEMS plots, approximate sample locations are annotated. For exact locations, see annotated supplementary figure 4, Supplementary Material online and supplementary table S1, Supplementary Material online. Features discussed in the main text and Supplementary Material online are labeled. *F*_ST_ values per panel emphasize the low absolute levels of differentiation.

In the broad overview Afro-Eurasia panel (fig. 1; *n* = 4,697 samples; 370 locales; *F*_ST_ = 0.071) we see that 19 out of 25 troughs visually align with plausible topographical obstacles to migration, such as deserts (Sahara; A1), seas (e.g., Mediterranean, Red, Black, Caspian, East China Seas; A2–8), marine straits (e.g., Mozambique Channel, Taiwan Strait; A9–10) and mountain ranges (Ural, Himalayas, Caucasus; A13, A11, middle of A3) or a combination thereof (e.g., the northeastern parts of A11, A12 roughly accord with the Tien Shan and the Tarim Basin, Altai and Gobi complex of mountains/desert, respectively). Many of these features, such as the Sahara desert (Cavalli-Sforza et al. 1994) or the Himalayas (Rosenberg et al. 2005; Bradburd et al. 2013) have been studied in great detail, as they are zones of not only genetic but also linguistic and ethnic differentiation. The remaining seven troughs (A19–A25) are found across Central Africa, Southern Africa, Scandinavia, and Siberia. In each of these regions, our sample consists of agricultural-based populations in relatively close proximity to traditionally hunter–gatherer or pastoralist populations. The island populations of the Andaman islands and New Guinea show troughs nearly contiguously around them (southern part of A11, and A15) reflecting their histories of relative isolation (Reich et al. 2009; Pugach et al. 2013). The other main features emerging at this scale are several large regions that have mostly high effective migration (such as within the European continent, the Arabian Peninsula, and East Asia).

Analyses on a finer geographic scale highlight subtler features (e.g., compare Europe in fig. 1 vs. fig. 2*a*), and reveal that differentiation exists on local and continental scales (supplementary table 2, Supplementary Material online). At these finer scales we continue to see troughs that align with landscape features, though increasingly we see troughs and corridors that coincide with contact zones of language groups and hypothesized areas of human migrations. For example, in Europe (fig. 2*b*) we observe troughs roughly in zones associated with language contact zones between Germanic and Northern Slavic speakers (W12) and between Northern Slavic speakers and the linguistically complex Caucasus region (W8). These, as well as most of the other features in Europe (troughs through the Alps, Adriatic, between Italy and Sardinia, in Northern Scandinavia), closely align with older results from classical markers (Barbujani and Sokal 1990). The Eastern Eurasian panel (fig. 2*e*) is largely consistent with the coarser-scale AEA panel. An exception is a corridor from Mongolia to the Caspian Sea (roughly E/W feature surrounded by E4–E7, E14, and E22), possibly reflecting genetic similarity over long distances brought about by the movements of Mongol and Turkic peoples, as the Kalmyk, Kazhaks, and Uygurs sample in this corridor all have well documented shared genetic ancestry with present-day populations of Southern Siberia and Mongolia (Yunusbayev et al. 2015). In Southeast Asia (fig. 2*h*), troughs align with several straits in the Malay archipelago (S6–S8). On the other hand, we observe two major corridors, one from Taiwan/Luzon through Western Mindanao to Sulawesi, and one from Ternate through the Lower Sunda Islands (LSI) into Melanesia. These could be a reflection of the Austronesian expansion that started roughly 3,000 years ago (Duggan and Stoneking 2014). In Africa (fig. 2*g*), a trough (A1) aligns with the Sahara desert and extends southeastward, roughly aligned with the language group boundaries between Niger-Congo and Afro-Asiatic language speakers (Campbell and Tishkoff 2008; supplementary fig. 7, Supplementary Material online). The West-African Afro-Asiatic speaking Hausa and Mada, together with the admixed Fulani (Bryc et al. 2010) show low effective migration to coastal West African Bantu speakers (A8). In Central Africa, corridors connecting West Africa with East and Southern Africa may reflect the Bantu expansion, and the Biaka and Mbuti show low effective migration (A7) with surrounding Bantu and Nilo-Saharan populations. In both Central and Eastern Africa, Nilo-Saharan and Niger-Congolese speakers overlap, resulting in low effective migration uncorrelated with language. Between Southern and Eastern Africans there is low effective migration through Mozambique and South-Western Tanzania (A4–A6). For a more detailed analysis, we constructed KhoeSan (SAKS, *n* = 109, 16 locales, *F*_ST_ = 0.025, fig. 2*k*) and Bantu (SAB, *n* = 30, 11 locales, *F*_ST_ = 0.014; fig. 2*l*) panels, which reveal very different spatial structuring. These results are broadly consistent with existing work on African population structure (Tishkoff et al. 2009; Bryc et al. 2010; Pickrell et al. 2012; Uren et al. 2016), and emphasize that African population structure appears largely determined by the Sahara desert, the Bantu and Arabic expansions, and the complex structure of hunter–gatherer groups specifically in South Africa.

We also contrasted the EEMS results to those obtained with principal component analysis (PCA). Although, PCA-biplots typically reflect large-scale gradients of diversity in a panel, EEMS emphasizes local distortions, such as troughs features that are often imperceptible in the PCA-biplots (fig. 1*b*; fig. 2*c,d,f,i,m*; supplementary fig. 6, Supplementary Material online). This is due, in part, to geographical information allowing EEMS to discern subtle structure while controlling for the effects of uneven sampling (Petkova et al. 2016), whereas the objective function of PCA minimizes the Frobenius-norm, and therefore emphasizes the largest pairwise genetic distances.

The maps we present provide compact summaries of the complex relationship of genes and geography in human populations. Most of the clearest features in these maps (e.g., the Alps, Sahara desert, Himalayas, W3, A1, E14; Nei and Roychoudhury 1993; Cavalli-Sforza et al. 1994; Bradburd et al. 2013) have been described previously and many represent regions where genetic, geographic, linguistic and ethnic differentiation all coincide. A subset of the trough features align with differences in subsistence strategies. Overall, the maps provided here support many previous inferences, typically made from more limited data sets, and provide an expanded demonstration of how human genetic diversity can reflect physical and cultural geography.

In contrast to methods that identify short bursts of gene flow (“admixture”) between diverged populations (Patterson et al. 2012; Loh et al. 2013; Hellenthal et al. 2014), EEMS models local migration between nearby groups to represent heterogeneous isolation-by-distance patterns. This leads to a few limitations that must be considered in interpretation: First, spatially heterogeneous isolation-by-distance is a flexible model, but not necessarily flexible enough to capture the complexity of human histories. For instance, human groups often overlap spatially while maintaining differentiation or have undergone long-distance migration/admixture not included in our model. These latter cases can produce geographic “outliers” that are difficult for EEMS to model. A clear example is Madagascar in the large AEA panel, which in the PCA is shifted toward samples from S.E. Asia (fig. 2*a*), presumably because of admixture from S.E. Asia to Madagascar (Kusuma et al. 2016). We found that running EEMS at high resolutions results in more interpretable plots as the surfaces can often accommodate modeling these samples within regions of relative isolation (e.g., A3 in the AFR panel models the differentiation of Madagascar from mainland samples, fig. 2*g*).

Second, decisions regarding which samples to include will affect the outcome of any analysis. When there is a feature inferred in a region with few samples, the exact positioning of the inferred change on the map will be imprecise (e.g., W4 in fig. 2*b*, presumably associated with the English Channel). The maps of posterior variance (supplementary figs. 2 and 4, Supplementary Material online) partly convey where there is uncertainty in positioning, but caution is still warranted as violations of the modeling assumptions will introduce further uncertainty. In other cases, the presence or absence of a particular group may impact the inference of corridors, sometimes depending on resolution. One example is the Kalmyk, a Mongolian people in Southern Russia. The Kalmyk are linked by a corridor to Mongolia (area surrounded by E22) in the CEA, but not the AEA panel; this corridor disappears in the CEA panel if the Kalmyks are excluded. Similarly, including the Eastern African Hadza and Sandawe (two language isolates) causes inference of a trough (eastern part of A1). This trough is broken up when we exclude these two samples. Another concern is that we merged data from studies whose sample inclusion criteria differ (e.g., four-grandparents from a single region vs. self-reported individual origin); however, based on exploratory analyses and the large spatial-scales treated here, we suspect these differences have minor effects on the overall landscapes inferred.

Third, the scales of the effective migration rates need to be interpreted with care. In each of our analysis panels, the absolute levels of differentiation are consistently low across all populations. EEMS draws attention to where differentiation is slightly elevated or depressed relative to expectations from geographic distance. Low effective migration between a pair of populations does not imply a complete absence of migration nor large levels of absolute differentiation; conversely, high levels of effective migration do not imply present-day ongoing gene flow. The EEMS surface is best understood as a modeling construct to visualize a relationship between genes and geography that is nonuniform across space. In particular, the emergence of migration features in the EEMS maps often align with known topography, past historical migrations, and/or linguistic/cultural distributions, but this is not an assessment of a causal connection. Formally testing the influence of specific features and environmental variables on migration rates remain important future tasks that will require extending EEMS or using different frameworks (Hanks and Hooten 2013).

Finally, it is worth reiterating the maps inferred here represent a model of gene flow that predicts genetic diversity in humans sampled today—a fuller representation would represent genetic structure dynamically through time. This is especially relevant as ancient DNA data have recently suggested human population structure can be surprisingly dynamic (e.g., Lazaridis et al. 2014). We suspect that some of the corridors are revealing elevated genetic similarity that has arisen from major gene flow events (e.g., in the AEA analysis, the connectivity through the Pontic Caspian Steppe may reflect the Bronze Age “Steppe” expansions inferred by Allentoft et al. 2015; Haak et al. 2015).

Overall, our migration landscapes suggest an alternative perspective from the clusters versus clines paradigms for human structure (Rosenberg et al. 2002; Serre and Pääbo 2004; Rosenberg et al. 2005): By revealing both sharp and diffuse features that structure human genetic diversity, our results suggest that more continuous definitions of ancestry in human population genetics can complement principal component methods or models of discrete populations with admixture. The results also help develop a more thorough geographic understanding of human genetic variation and its distribution. For instance, as rare variants are often geographically localized (Gibson 2012; Mathieson and McVean 2012), the maps presented here may be especially useful for predicting ancestries within which rare alleles (some of which will have medical relevance) might be contained. The maps also annotate features of present-day population structure that ancient DNA and historical/archaeological studies can aim to explain.

## Materials and Methods

### Merging Genetic Data

We obtained SNP genotype data from 27 different studies (supplementary table 1, Supplementary Material online). Processing was done using a reproducible snakemake pipeline (Köster and Rahmann 2012) available under http://github.com/NovembreLab/eems-merge, heavily relying on plink 1.9 (Chang et al. 2015) for handling genotypes. The sources differ in the input format and preprocessing, however in general we performed the following steps:

1. Remove all nonautosomal, non-SNP variants
2. Map SNPs to the forward strand of human reference genome b37 coordinates using chip manufacturer metadata files or SNP identifiers
3. Remove strand-ambiguous A/T and G/C variants

The remaining SNPs were then merged using successive plink –bmerge commands into a single master data set with 9,003 individuals and 1.9 M SNPs but a total genotyping rate of only 20.6%. Forty six SNPs were removed because different studies reported different alternative alleles. We used a relationship filter of 0.6 using the “–rel-cutoff 0.6” flag in plink to remove 667 closely related individuals or duplicates. After merging, each analysis panel had missingness rates <0.5% (AEA = 0.2%, WEA = 0.3%, CEA = 0.2%, SEA = 0.5%, AFR = 0.2%, SAHG = 0.1%). In all panels, all SNPs passed a one-sided HWE-test (*P*-value < 10^−5^), with the exception of SEA, where nine (out of 7,553 SNPs) failed and were excluded.

### Data Retrieval and Filtering

#### Human Origins Data Set

Sampling location information was obtained from table S9.4 of Lazaridis et al. (2014), and the data were shared by David Reich. We used the population information in the “vdata” subset of all ascertainment panels, except for the analysis where we assess ascertainment bias. The utility “convert” from “admixtools” (Patterson et al. 2012) was used to convert the data into plink format.

#### Estonian Biocentre Data

The data generated by the Estonian Biocentre (Behar et al. 2013; Cardona et al. 2014; Chaubey et al. 2011; Di Cristofaro et al. 2013; Fedorova et al. 2013; Kovacevic et al. 2014; Metspalu et al. 2011; Migliano et al. 2013; Pierron et al. 2014; Raghavan et al. 2014; Rasmussen et al. 2010, 2011; Skoglund et al. 2014; Yunusbayev et al. 2012, 2015) were provided in plink format by Mait Metspalu on October 30, 2015, along with location information where it was available. This data set contained 1,282,568 SNPs. Of those, 6,770 SNPs had nonunique ids and were removed.

#### HUGO Pan-Asian SNP Consortium

The data were downloaded on June 24, 2015 from www.biotec.or.th/PASNP (HUGO Pan-Asian SNP Consortium 2009). Location-metadata were obtained on the same day from the map on the same website, and individuals were matched to populations using the individual identifiers. All individuals with the same tag were assigned the median of all locations from that tag. The data were first lifted onto hg19 (with 5 out of 54,794 SNPs being removed), and then reformatted into binary plink format. Because of the small size of the chip used and the low overlap with the human origins array in particular, we only consider this data in the Southeast Asian panel.

#### Uniform Global Sample

This data were downloaded on June 20, 2015 from http://jorde-lab.genetics.utah.edu/pub/affy6_xing2010/ (Xing et al. 2010). Sampling locations were provided by Jinchuan Xing. We used version 32 of the annotation file obtained on June 19, 2015 from affymetrix.com to map SNPs onto hg19, remove strand-ambiguous SNPs and to flip SNPs that were on the minus-strand.

#### POPRES Data

POPRES data were obtained under dbGAP study accession phs000145 to John Novembre, and we used the data as processed in Novembre et al. (2008), and only retain individuals for which all grandparents were from the same country, and labeled the Swiss sample according to self-reported language (Nelson et al. 2008). We used version 32 of the annotation file obtained on June 19, 2015 from www.affymetrix.com (“Mapping250K_sp.na32.annot.csv” and “Mapping250K_Sty.na32.annot.csv”) to filter SNPs that did not map onto hg19 and we removed strand-ambiguous AT and GC polymorphisms.

#### African Data

Data from Bryc et al. (2009) and Hunter-Zinck et al. (2010) were obtained on April 19, 2017 from David Comas’ website under http://www.biologiaevolutiva.org/dcomas/? p=607. We used version 32 of the annotation file GenomeWideSNP_6.na32.annot.csv” obtained on June 19, 2015 from affymetrix.com to map SNPs onto hg19, remove strand-ambiguous SNPs and to flip SNPs that were on the minus-strand.

#### Southeast Asian Data

The data were obtained on July 14, 2015 from Mark Stoneking in three different source files (Reich et al. 2011). After merging the three different source files, SNPs not mapping to hg19 using the annotation file “GenomeWideSNP_6.na32.annot.csv” were removed, as were AT and GC SNPs. Sampling locations were extracted from figure 1 of Reich et al. (2011).

#### Mediterranean Panel

Data were obtained on August 13, 2015 in binary plink format from http://drineas.org/Maritime_Route/RAW_DATA/PLINK_FILES/MARITIME_ROUTE.zip (Paschou et al. 2014). Sampling location information was obtained from supplementary table 3 in Paschou et al. (2014). SNPs not mapping to hg19 using the annotation file “GenomeWideSNP_6.na32.annot.csv” were removed, as were AT and GC SNPs.

#### Tibetan and Himalayan Data

Data from Bigham et al. (2010), Xu et al. (2011), and Jeong et al. (2017) were obtained from Choongwon Jeong and Anna Di Rienzo. We used the same filtering as in the Jeong et al. (2017) study, but only added the samples originating from these three studies with permission from the respective authors.

### Combining Meta-Information

All sources with the exception of the Estonian Biocentre data provided (approximate) sampling coordinates. However, the level of accuracy varied between sources, with some providing specific ethnicities, some (such as POPRES) only providing country information and others just providing city- or state-level information. For POPRES-derived data, and most countries, we assigned individuals to the country’s centerpoint, with the exception of Sweden and Finland, which were assigned their capital. For the Estonian Biocentre data, sampling location data were highly heterogeneous. Samples that could not be confidently assigned to a region with an accuracy of 100 km were excluded. For populations with samples from multiple studies, the most accurate source location was used. For locations covered with different accuracy, only the most accurate samples were retained. For example, we dropped all Spanish individuals from POPRES (only country level data), as the Human Origins data provided higher resolution, with samples from eleven different regions in Spain. The resulting table is given as supplementary table S1, Supplementary Material online.

#### Language Data

To validate troughs correlating with presumed language barriers, we cross-referenced the genetic data with linguistic data from the Glottolog 3.2 database (Hammarström et al. 2018). To do so, we compared the correlation of pairwise genetic distance and geographic distances within and between pairs of language groups. As there was frequently no primary data recording the language of speakers, we proceeded as follows: For population identifiers that correspond to languages/or ethnic groups with a clear majority language, we used that language. For samples with country-level information where the country has a clear majority language (e.g., Germany, Slovenia), that language was assigned (supplementary table S1, Supplementary Material online). Otherwise, if a sample was from a region with a clear majority language that is not obviously due to recent colonization, that language was assigned. All other samples were not assigned a language. For simplicity, we group Nilotic, Central Sudanic, and Mande languages into “Nilo-Saharan,” Khoe, Kxa, and Tuu speakers into “KhoeSan” and Armenic, Circassian, Kartvelian, and Nakh-Daghesanian into “Caucasus.” For all troughs, we test the hypothesis that they align with boundaries between linguistic groups, by performing a partial mantel test comparing genetic distances and language groups as a categorical variable using the implementation in the R-package “vegan”(Oksanen et al. 2007). We note that results need to be interpreted cautiously, as the mantel test is generally poorly calibrated for spatially autocorrelated data (Guillot and Rousset 2013).

#### Samples Omitted from Model Fitting

Besides samples whose geographic origin we could not unambiguously assign (*n* = 74), we removed a small number of samples that would violate some assumptions of the EEMS model. In particular, we excluded all Jewish samples (*n* = 379), due to complexity of the diaspora and subsequent local admixture (Behar et al. 2010) and Han-Chinese in Taiwan and Singapore (*n* = 170), who both are recent migrant population to those locales. To avoid any possible distortion due to uneven sampling, we downsampled all single locales to at most 50 individuals, drawn independently for different panels. This resulted in a total of 6,066 individuals used in at least one panel (supplementary table S1, Supplementary Material online).

### Visualization Pipeline

We developed a second pipeline using snakemake (Köster and Rahmann 2012) to perform all subsetting and demographic analyses, available under github.com/NovembreLab/eems-around-the-world. The pipeline allows for defining panels using a flexible set of features, including latitudinal and longitudinal boundaries, continent or country of samples, source study, as well as the addition and exclusion of particular samples or populations. Based on these subsets, different modules allow performing EEMS and PCA analyses, as well as generating all the figures, that were then annotated using the software Inkscape (http://inkscape.org; last accessed December 9, 2019). All configuration variables are stored in json and yaml config files. We perform EEMS and PCA for each panel independently. Structural variants are a potential confounding factor for genome-wide SNP based analysis. In PCA, these variants may result in a number of neighboring SNP in high LD to have very high loadings, thus overemphasizing the effect of these variants. For this reason, it is advisable to remove regions containing SNP that have extremely high loadings on some principal component. Thus, for each panel, we perform a preliminary PCA analysis using flashpca (Abraham and Inouye 2014). The loading-scores for each PC were normalized by dividing them by the standard deviations on each PC [outlier_score = L[i]/sd(L[i])], and then we removed a 200 kb window around any SNP for which |outlier_score| > 5. We also dropped individuals with >5% missingness, and SNPs with >1% missing data from each panel.

#### EEMS

To generate the map surfaces with EEMS (https://github.com/dipetkov/eems), we must choose a grid size and boundaries. Choosing a coarse grid results in faster computation, but only produces a map with broad-scale patterns. A finer grid, on the other hand, is able to reveal more details, but at a steep increase in computational cost and with an increased danger of introducing patterns that are harder to interpret. Grid density and sizes are given in supplementary table 1, Supplementary Material online, along with population level *F*_ST_ calculated using plink, and *F*_ST_ based on the mean migration rate inferred by EEMS and equilibrium stepping stone model theory (Slatkin 1991).

We evaluated the impact of SNP ascertainment bias by running EEMS on the multiple, documented SNP ascertainment panels of the Human Origins data (Lazaridis et al. 2014). We found that while ascertainment bias has an effect on the heterozygosity surfaces that EEMS estimates, the migration surfaces remain relatively unaffected (supplementary fig. 1, Supplementary Material online). Therefore, we restrict our presentation to the migration surfaces.

EEMS approximates a continuous region with a triangular grid, which has to be specified. We generated global geodesic graphs at three resolutions (approximate distance between demes of 120, 240, and 500 km, respectively) using dggrid v6.1 (Sahr et al. 2003) and intersected these graphs with the area representing each panel (figs. 1 and 2). For each panel, we performed four pilot runs of 2–8 million iterations each. The run with the highest likelihood was then used for a second set of four runs of 4–10 million iteration each, with the first 500,000 discarded as burn-in. Number of iterations were chosen such that the total computation time per single run was around 10 days. Every 20,000th iteration was sampled. All other (hyper-)parameters were kept at their default values (Petkova et al. 2016). We compared EEMS to an isolation-by-distance model with a constant migration rate by refitting EEMS allowing only a single migration rate tile, but arbitrary diversity rate tiles using the otherwise same settings. The resulting log Bayes factors are given in supplementary table 2, Supplementary Material online.

#### Evaluating Fit of EEMS and PCA to Genetic Distances

For EEMS, the posterior samples imply an expected distance matrix between populations. For PCA, the components and their loadings provide an approximation to the genetic distance matrix between individuals. We use the median PCA values of individuals across two, ten, or 100 PC components to produce an expected genetic distance matrix between populations. For each method, the expected genetic distance matrices are compared with the observed matrices using a simple linear correlation computed between all pairwise distances.

## Supplementary Material

msz280_Supplementary_Data

## References

[msz280-B1] Abraham G, Inouye M. 2014 Fast principal component analysis of large-scale genome-wide data. PLoS One 9(4):e93766. 24718290 10.1371/journal.pone.0093766 PMC3981753

[msz280-B2] Allentoft ME, Sikora M, Sjögren K-G, Rasmussen S, Rasmussen M, Stenderup J, Damgaard PB, Schroeder H, Ahlström T, Vinner L, et al 2015 Population genomics of Bronze Age Eurasia. Nature 522(7555):167–172. 26062507 10.1038/nature14507

[msz280-B3] Barbujani G, Sokal RR. 1990 Zones of sharp genetic change in Europe are also linguistic boundaries. Proc Natl Acad Sci USA. 87(5):1816–1819. 2308939 10.1073/pnas.87.5.1816 PMC53574

[msz280-B4] Battey CJ, Ralph PL, Kern AD. 2019. Space is the place: effects of continuous spatial structure on analysis of population genetic data. bioRxiv 659235; doi: https://doi.org/10.1101/659235 10.1534/genetics.120.303143 PMC7198281 32209569

[msz280-B5] Behar DM, Metspalu M, Baran Y, Kopelman NM, Yunusbayev B, Gladstein A, Tzur S, Sahakyan H, Bahmanimehr A, Yepiskoposyan L, et al 2013 No evidence from genome-wide data of a Khazar origin for the Ashkenazi Jews. Hum Biol. 85(6):859–900. 25079123 10.3378/027.085.0604

[msz280-B6] Behar DM, Yunusbayev B, Metspalu M, Metspalu E, Rosset S, Parik J, Rootsi S, Chaubey G, Kutuev I, Yudkovsky G, et al 2010 The genome-wide structure of the Jewish people. Nature 466(7303):238–242. 20531471 10.1038/nature09103

[msz280-B7] Bigham A, Bauchet M, Pinto D, Mao X, Akey JM, Mei R, Scherer SW, Julian CG, Wilson MJ, López Herráez D, et al 2010 Identifying signatures of natural selection in Tibetan and Andean populations using dense genome scan data. PLoS Genet. 6(9):e1001116. 20838600 10.1371/journal.pgen.1001116 PMC2936536

[msz280-B8] Bradburd GS, Coop GM, Ralph PL. 2018 Inferring continuous and discrete population genetic structure across space. Genetics [Internet] 210(1):33–52. 10.1534/genetics.118.301333 PMC6116973 30026187

[msz280-B9] Bradburd GS, Ralph PL. 2019 Spatial population genetics: it's about time. *Annu Rev Ecol Evol Syst* 50:427–449. https://doi.org/10.1146/annurev-ecolsys-110316-022659.

[msz280-B10] Bradburd GS, Ralph PL, Coop GM. 2013 Disentangling the effects of geographic and ecological isolation on genetic differentiation. Evolution 67(11):3258–3273. 24102455 10.1111/evo.12193 PMC3808528

[msz280-B11] Bradburd GS, Ralph PL, Coop GM. 2016 A spatial framework for understanding population structure and admixture. PLoS Genet. 12(1):e1005703. 26771578 10.1371/journal.pgen.1005703 PMC4714911

[msz280-B12] Bryc K, Auton A, Nelson MR, Oksenberg JR, Hauser SL, Williams S, Froment A, Bodo J-M, Wambebe C, Tishkoff SA, et al 2009 Genome-wide patterns of population structure and admixture in West Africans and African Americans. Proc Natl Acad Sci USA [Internet]. Available from: http://www.pnas.org/content/early/2009/12/10/0909559107 10.1073/pnas.0909559107 PMC2818934 20080753

[msz280-B13] Bryc K, Auton A, Nelson MR, Oksenberg JR, Hauser SL, Williams S, Froment A, Bodo J-M, Wambebe C, Tishkoff SA, et al 2010 Genome-wide patterns of population structure and admixture in West Africans and African Americans. Proc Natl Acad Sci USA. 107(2):786–791. 20080753 10.1073/pnas.0909559107 PMC2818934

[msz280-B14] Campbell MC, Tishkoff SA. 2008 African genetic diversity: implications for human demographic history, modern human origins, and complex disease mapping. Annu Rev Genom Hum Genet. 9(1):403–433. 10.1146/annurev.genom.9.081307.164258 PMC2953791 18593304

[msz280-B15] Cardona A, Pagani L, Antao T, Lawson DJ, Eichstaedt CA, Yngvadottir B, Shwe MTT, Wee J, Romero IG, Raj S, et al 2014 Genome-wide analysis of cold adaptation in indigenous Siberian populations. PLoS One 9(5):e98076. 24847810 10.1371/journal.pone.0098076 PMC4029955

[msz280-B16] Cavalli-Sforza LL, Menozzi P, Piazza A. 1994 The history and geography of human genes. Princeton NJ, USA: Princeton University Press.

[msz280-B17] Chang CC, Chow CC, Tellier LC, Vattikuti S, Purcell SM, Lee JJ. 2015 Second-generation PLINK: rising to the challenge of larger and richer datasets. GigaScience 4(1):7. 25722852 10.1186/s13742-015-0047-8 PMC4342193

[msz280-B18] Chaubey G, Metspalu M, Choi Y, Mägi R, Romero IG, Soares P, van OM, Behar DM, Rootsi S, Hudjashov G, et al 2011 Population genetic structure in Indian Austroasiatic speakers: the role of landscape barriers and sex-specific admixture. Mol Biol Evol. 28(2):1013–1024. 20978040 10.1093/molbev/msq288 PMC3355372

[msz280-B19] Di Cristofaro J, Pennarun E, Mazières S, Myres NM, Lin AA, Temori SA, Metspalu M, Metspalu E, Witzel M, King RJ, et al 2013 Afghan Hindu Kush: where Eurasian sub-continent gene flows converge. PLoS One 8(10):e76748. 24204668 10.1371/journal.pone.0076748 PMC3799995

[msz280-B20] Duggan AT, Stoneking M. 2014 Recent developments in the genetic history of East Asia and Oceania. Curr Opin Genet Dev. 29:9–14. 25170982 10.1016/j.gde.2014.06.010

[msz280-B21] Fedorova SA, Reidla M, Metspalu E, Metspalu M, Rootsi S, Tambets K, Trofimova N, Zhadanov SI, Kashani BH, Olivieri A, et al 2013 Autosomal and uniparental portraits of the native populations of Sakha (Yakutia): implications for the peopling of Northeast Eurasia. BMC Evol Biol. 13(1):127. 23782551 10.1186/1471-2148-13-127 PMC3695835

[msz280-B22] Frantz AC, Cellina S, Krier A, Schley L, Burke T. 2009 Using spatial Bayesian methods to determine the genetic structure of a continuously distributed population: clusters or isolation by distance? J Appl Ecol [Internet]. 46(2):493–505.

[msz280-B23] Gibson G. 2012 Rare and common variants: twenty arguments. Nat Rev Genet. 13(2):135–145. 22251874 10.1038/nrg3118 PMC4408201

[msz280-B24] Guillot G, Leblois R, Coulon A, Frantz AC. 2009 Statistical methods in spatial genetics. Mol Ecol. 18(23):4734–4756. 19878454 10.1111/j.1365-294X.2009.04410.x

[msz280-B25] Guillot G, Rousset F. 2013 Dismantling the Mantel tests. Methods Ecol Evol. 4(4):336–344.

[msz280-B26] Haak W, Lazaridis I, Patterson N, Rohland N, Mallick S, Llamas B, Brandt G, Nordenfelt S, Harney E, Stewardson K, et al 2015 Massive migration from the steppe was a source for Indo-European languages in Europe. Nature 522(7555):207–211. 25731166 10.1038/nature14317 PMC5048219

[msz280-B27] Hammarström H, Bank S, Forkel R, Haspelmath M. 2018 Glottolog 3.2. https://glottolog.org/ last accessed December 9, 2019.

[msz280-B28] Hanks EM, Hooten MB. 2013 Circuit theory and model-based inference for landscape connectivity. J Am Stat Assoc. 108(501):22–33.

[msz280-B29] Hellenthal G, Busby GBJ, Band G, Wilson JF, Capelli C, Falush D, Myers S. 2014 A genetic atlas of human admixture history. Science 343(6172):747–751. 24531965 10.1126/science.1243518 PMC4209567

[msz280-B30] House GL, Hahn MW. 2018 Evaluating methods to visualize patterns of genetic differentiation on a landscape. Mol Ecol Resour. 18(3):448–460. 29282875 10.1111/1755-0998.12747

[msz280-B31] HUGO Pan-Asian SNP Consortium. 2009 Mapping human genetic diversity in Asia. Science 326:1541–1545. 20007900 10.1126/science.1177074

[msz280-B32] Hunter-Zinck H, Musharoff S, Salit J, Al-Ali KA, Chouchane L, Gohar A, Matthews R, Butler MW, Fuller J, Hackett NR, et al 2010 Population genetic structure of the people of Qatar. Am J Hum Genet. 87(1):17–25. 20579625 10.1016/j.ajhg.2010.05.018 PMC2896773

[msz280-B33] Jeong C, Peter BM, Basnyat B, Neupane M, Beall CM, Childs G, Craig SR, Novembre J, Di Rienzo A. 2017 A longitudinal cline characterizes the genetic structure of human populations in the Tibetan plateau. PLoS One 12(4):e0175885. 28448508 10.1371/journal.pone.0175885 PMC5407838

[msz280-B34] Köster J, Rahmann S. 2012 Snakemake—a scalable bioinformatics workflow engine. Bioinformatics 28:2520–2522. 22908215 10.1093/bioinformatics/bts480

[msz280-B35] Kovacevic L, Tambets K, Ilumäe A-M, Kushniarevich A, Yunusbayev B, Solnik A, Bego T, Primorac D, Skaro V, Leskovac A, et al 2014 Standing at the gateway to Europe—the genetic structure of Western Balkan populations based on autosomal and haploid markers. PLoS One 9(8):e105090. 25148043 10.1371/journal.pone.0105090 PMC4141785

[msz280-B36] Kusuma P, Brucato N, Cox MP, Pierron D, Razafindrazaka H, Adelaar A, Sudoyo H, Letellier T, Ricaut F-X. 2016 Contrasting linguistic and genetic origins of the Asian source populations of Malagasy. Sci Rep. 6:26066. 27188237 10.1038/srep26066 PMC4870696

[msz280-B37] Lazaridis I, Patterson N, Mittnik A, Renaud G, Mallick S, Kirsanow K, Sudmant PH, Schraiber JG, Castellano S, Lipson M, et al 2014 Ancient human genomes suggest three ancestral populations for present-day Europeans. Nature 513(7518):409–413. 25230663 10.1038/nature13673 PMC4170574

[msz280-B38] Loh P-R, Lipson M, Patterson N, Moorjani P, Pickrell JK, Reich D, Berger B. 2013 Inferring admixture histories of human populations using linkage disequilibrium. Genetics 193(4):1233–1254. 23410830 10.1534/genetics.112.147330 PMC3606100

[msz280-B39] Mathieson I, McVean G. 2012 Differential confounding of rare and common variants in spatially structured populations. Nat Genet. 44(3):243–246. 22306651 10.1038/ng.1074 PMC3303124

[msz280-B40] Metspalu M, Romero IG, Yunusbayev B, Chaubey G, Mallick CB, Hudjashov G, Nelis M, Mägi R, Metspalu E, Remm M, et al 2011 Shared and unique components of human population structure and genome-wide signals of positive selection in South Asia. Am J Hum Genet. 89(6):731–744. 22152676 10.1016/j.ajhg.2011.11.010 PMC3234374

[msz280-B41] Migliano A, Romero I, Metspalu M, Leavesley M, Pagani L, Antao T, Huang D-W, Sherman B, Siddle K, Scholes C, et al 2013 Evolution of the pygmy phenotype: evidence of positive selection from genome-wide scans in African, Asian, and Melanesian pygmies. Hum Biol [Internet]. 85:251–284. Available from: http://digitalcommons.wayne.edu/humbiol/vol85/iss1/12 10.3378/027.085.0313 24297229

[msz280-B42] Nei M, Roychoudhury AK. 1993 Evolutionary relationships of human populations on a global scale. Mol Biol Evol. 10(5):927–943. 8412653 10.1093/oxfordjournals.molbev.a040059

[msz280-B43] Nelson MR, Bryc K, King KS, Indap A, Boyko AR, Novembre J, Briley LP, Maruyama Y, Waterworth DM, Waeber G, et al 2008 The Population Reference Sample, POPRES: a resource for population, disease, and pharmacological genetics research. Am J Hum Genet. 83(3):347–358. 18760391 10.1016/j.ajhg.2008.08.005 PMC2556436

[msz280-B44] Novembre J, Johnson T, Bryc K, Kutalik Z, Boyko AR, Auton A, Indap A, King KS, Bergmann S, Nelson MR, et al 2008 Genes mirror geography within Europe. Nature 456(7218):98–101. 18758442 10.1038/nature07331 PMC2735096

[msz280-B45] Novembre J, Peter BM. 2016 Recent advances in the study of fine-scale population structure in humans. Curr Opin Genet Dev. 41:98–105. 27662060 10.1016/j.gde.2016.08.007 PMC5291306

[msz280-B46] Oksanen J, Kindt R, Legendre P, O’Hara B, Stevens MHH, Oksanen MJ, Suggests M. 2007 The vegan package. Commun Ecol Pack. 10:631–637.

[msz280-B47] Paschou P, Drineas P, Yannaki E, Razou A, Kanaki K, Tsetsos F, Padmanabhuni SS, Michalodimitrakis M, Renda MC, Pavlovic S, et al 2014 Maritime route of colonization of Europe. Proc Natl Acad Sci USA. 111(25):9211–9216. 24927591 10.1073/pnas.1320811111 PMC4078858

[msz280-B48] Patterson NJ, Moorjani P, Luo Y, Mallick S, Rohland N, Zhan Y, Genschoreck T, Webster T, Reich D. 2012 Ancient admixture in human history. Genetics 192(3):1065. 22960212 10.1534/genetics.112.145037 PMC3522152

[msz280-B49] Perez MF, Franco FF, Bombonato JR, Bonatelli IAS, Khan G, Romeiro-Brito M, Fegies AC, Ribeiro PM, Silva GAR, Moraes EM. 2018 Assessing population structure in the face of isolation by distance: are we neglecting the problem? Divers Distrib. 24(12):1883–1889.

[msz280-B50] Petkova D, Novembre J, Stephens M. 2016 Visualizing spatial population structure with estimated effective migration surfaces. Nat Genet. 48(1):94–100. 26642242 10.1038/ng.3464 PMC4696895

[msz280-B51] Pickrell JK, Patterson N, Barbieri C, Berthold F, Gerlach L, Güldemann T, Kure B, Mpoloka SW, Nakagawa H, Naumann C, et al 2012 The genetic prehistory of southern Africa. Nat Commun. 3:1143. 23072811 10.1038/ncomms2140 PMC3493647

[msz280-B52] Pierron D, Razafindrazaka H, Pagani L, Ricaut F-X, Antao T, Capredon M, Sambo C, Radimilahy C, Rakotoarisoa J-A, Blench RM, et al 2014 Genome-wide evidence of Austronesian–Bantu admixture and cultural reversion in a hunter–gatherer group of Madagascar. Proc Natl Acad Sci USA. 111(3):936–941. 24395773 10.1073/pnas.1321860111 PMC3903192

[msz280-B53] Pugach I, Delfin F, Gunnarsdóttir E, Kayser M, Stoneking M. 2013 Genome-wide data substantiate Holocene gene flow from India to Australia. Proc Natl Acad Sci USA. 110(5):1803–1808. 23319617 10.1073/pnas.1211927110 PMC3562786

[msz280-B54] Raghavan M, Skoglund P, Graf KE, Metspalu M, Albrechtsen A, Moltke I, Rasmussen S, Stafford TW Jr, Orlando L, Metspalu E, et al 2014 Upper Palaeolithic Siberian genome reveals dual ancestry of Native Americans. Nature 505(7481):87–91. 24256729 10.1038/nature12736 PMC4105016

[msz280-B55] Ramachandran S, Deshpande O, Roseman CC, Rosenberg NA, Feldman MW, Cavalli-Sforza LL. 2005 Support from the relationship of genetic and geographic distance in human populations for a serial founder effect originating in Africa. Proc Natl Acad Sci USA. 102(44):15942–15947. 16243969 10.1073/pnas.0507611102 PMC1276087

[msz280-B56] Rasmussen M, Guo X, Wang Y, Lohmueller KE, Rasmussen S, Albrechtsen A, Skotte L, Lindgreen S, Metspalu M, Jombart T, et al 2011 An aboriginal Australian genome reveals separate human dispersals into Asia. Science 334(6052):94–98. 21940856 10.1126/science.1211177 PMC3991479

[msz280-B57] Rasmussen M, Li Y, Lindgreen S, Pedersen JS, Albrechtsen A, Moltke I, Metspalu M, Metspalu E, Kivisild T, Gupta R, et al 2010 Ancient human genome sequence of an extinct Palaeo-Eskimo. Nature 463(7282):757–762. 20148029 10.1038/nature08835 PMC3951495

[msz280-B59] Reich D, Patterson N, Kircher M, Delfin F, Nandineni MR, Pugach I, Ko A-S, Ko Y-C, Jinam TA, Phipps ME, et al 2011 Denisova admixture and the first modern human dispersals into Southeast Asia and Oceania. Am J Hum Genet. 89(4):516–528. 21944045 10.1016/j.ajhg.2011.09.005 PMC3188841

[msz280-B60] Reich D, Thangaraj K, Patterson N, Price AL, Singh L. 2009 Reconstructing Indian population history. Nature 461(7263):489–494. 19779445 10.1038/nature08365 PMC2842210

[msz280-B61] Ringbauer H, Coop G, Barton NH. 2017 Inferring recent demography from isolation by distance of long shared sequence blocks. *Genetics*, 205(3):1335–1351. 10.1534/genetics.116.196220 PMC5340342 28108588

[msz280-B62] Ringbauer H, Kolesnikov A, Field DL, Barton NH. 2018 Estimating barriers to gene flow from distorted isolation-by-distance patterns. Genetics 208(3):1231–1245. 29311149 10.1534/genetics.117.300638 PMC5844333

[msz280-B63] Rosenberg NA, Mahajan S, Ramachandran S, Zhao C, Pritchard JK, Feldman MW. 2005 Clines, clusters, and the effect of study design on the inference of human population structure. PLoS Genet. 1(6):e70. 16355252 10.1371/journal.pgen.0010070 PMC1310579

[msz280-B64] Rosenberg NA, Pritchard JK, Weber JL, Cann HM, Kidd KK, Zhivotovsky LA, Feldman MW. 2002 Genetic structure of human populations. Science 298(5602):2381–2385. 12493913 10.1126/science.1078311

[msz280-B65] Sahr K, White D, Kimerling AJ. 2003 Geodesic discrete global grid systems. Cartogr Geogr Inf Sci. 30(2):121–134.

[msz280-B66] Serre D, Pääbo S. 2004 Evidence for gradients of human genetic diversity within and among continents. Genome Res. 14(9):1679–1685. 15342553 10.1101/gr.2529604 PMC515312

[msz280-B67] Skoglund P, Malmström H, Omrak A, Raghavan M, Valdiosera C, Günther T, Hall P, Tambets K, Parik J, Sjögren K-G, et al 2014 Genomic diversity and admixture differs for stone-age Scandinavian foragers and farmers. Science 344(6185):747–750. 24762536 10.1126/science.1253448

[msz280-B68] Slatkin M. 1991 Inbreeding coefficients and coalescence times. Genet Res. 58(2):167–175. 1765264 10.1017/s0016672300029827

[msz280-B69] Tishkoff SA, Reed FA, Friedlaender FR, Ehret C, Ranciaro A, Froment A, Hirbo JB, Awomoyi AA, Bodo J-M, Doumbo O, et al 2009 The genetic structure and history of Africans and African Americans. Science 324(5930):1035–1044. 19407144 10.1126/science.1172257 PMC2947357

[msz280-B70] Uren C, Kim M, Martin AR, Bobo D, Gignoux CR, van Helden PD, Möller M, Hoal EG, Henn BM. 2016 Fine-scale human population structure in Southern Africa reflects ecogeographic boundaries. Genetics 204(1):303–314. 27474727 10.1534/genetics.116.187369 PMC5012395

[msz280-B71] Wang C, Zöllner S, Rosenberg NA. 2012 A quantitative comparison of the similarity between genes and geography in worldwide human populations. PLoS Genet. 8(8):e1002886. 22927824 10.1371/journal.pgen.1002886 PMC3426559

[msz280-B72] Xing J, Watkins WS, Shlien A, Walker E, Huff CD, Witherspoon DJ, Zhang Y, Simonson TS, Weiss RB, Schiffman JD, et al 2010 Toward a more uniform sampling of human genetic diversity: a survey of worldwide populations by high-density genotyping. Genomics 96(4):199–210. 20643205 10.1016/j.ygeno.2010.07.004 PMC2945611

[msz280-B73] Xu S, Li S, Yang Y, Tan J, Lou H, Jin W, Yang L, Pan X, Wang J, Shen Y, et al 2011 A genome-wide search for signals of high-altitude adaptation in Tibetans. Mol Biol Evol. 28(2):1003–1011. 20961960 10.1093/molbev/msq277

[msz280-B74] Yunusbayev B, Metspalu M, Järve M, Kutuev I, Rootsi S, Metspalu E, Behar DM, Varendi K, Sahakyan H, Khusainova R, et al 2012 The Caucasus as an asymmetric Semipermeable barrier to ancient human migrations. Mol Biol Evol. 29(1):359–365. 21917723 10.1093/molbev/msr221

[msz280-B75] Yunusbayev B, Metspalu M, Metspalu E, Valeev A, Litvinov S, Valiev R, Akhmetova V, Balanovska E, Balanovsky O, Turdikulova S, et al 2015 The genetic legacy of the expansion of Turkic-speaking nomads across Eurasia. PLoS Genet. 11(4):e1005068. 25898006 10.1371/journal.pgen.1005068 PMC4405460

