# Supplementary material for: Genetic Landscapes Reveal How Human Genetic Diversity Aligns with Geography
Source: Mol Biol Evol. 2019 Nov 28;37(4):943–51. doi: 10.1093/molbev/msz280 (PMC7086171; doi:10.1093/molbev/msz280)
Supplement: msz280_Supplementary_Data [file msz280_supplementary_data.pdf]

Supplemental Data

Study	Abbrev.	Samples	Locations	Source
Bryc et al. 2009	B09	121	11	(Bryc et al. 2009)
Behar et al. 2010	Be10	295	22	(Behar et al. 2010)https://paperpile.com/c/qxd37D/vJFc
Behar et al. 2013	B13	131	20	(Behar et al. 2013)https://paperpile.com/c/qxd37D/XuSP
Bigham et al. 2010	Bi10	45	3	(Bigham et al. 2010)https://paperpile.com/c/qxd37D/teA7
Chaubey et al. 2011	C11	37	5	(Chaubey et al. 2011)https://paperpile.com/c/qxd37D/nnfC
Cardona et al. 2014	C14	192	37	(Cardona et al. 2014)https://paperpile.com/c/qxd37D/jTHh
Di Cristofaro et al. 2013	D13	14	3	(Di Cristofaro et al. 2013)https://paperpile.com/c/qxd37D/YmmK
Fedorova et al. 2013	F13	30	6	(Fedorova et al. 2013)https://paperpile.com/c/qxd37D/QICB
HUGO Consortium 2009	H09	975	47	(HUGO Pan-Asian SNP Consortium 2009)https://paperpile.com/c/qxd37D/ImE4
Hunter-Zinck et al. 2010	H10	85	1	(Hunter-Zinck et al. 2010)
Jeong et al. 2017	J17	53	2	(Jeong et al. 2017)
Kovacevic et al. 2014	K14	70	6	(Kovacevic et al. 2014)https://paperpile.com/c/qxd37D/TRPG
Lazaridis et al. 2014	L14	1590	159	(Lazaridis, Patterson, Mittnik, Renaud, Mallick, et al. 2014)https://paperpile.com/c/qxd37D/v7MN
Metspalu et al. 2011	M11	127	11	(Metspalu et al. 2011)https://paperpile.com/c/qxd37D/jvhx
Migliano et al. 2013	M13	68	6	(Migliano et al. 2013)https://paperpile.com/c/qxd37D/wY1j
Nelson et al. 2008	N08	531	29	(Nelson et al. 2008)https://paperpile.com/c/qxd37D/wpE1
Paschou et al. 2014	Pa14	626	29	(Paschou et al. 2014)https://paperpile.com/c/qxd37D/Zj9t
Pierron et al. 2014	Pi14	114	5	(Pierron et al. 2014)https://paperpile.com/c/qxd37D/XG3v
Raghavan et al. 2014	R14	83	9	(Raghavan et al. 2014)https://paperpile.com/c/qxd37D/LoVd
Rasmussen et al. 2010	R10	101	9	(Rasmussen et al. 2010)https://paperpile.com/c/qxd37D/SMSC
Rasmussen et al. 2011	Ra11	19	3	(Rasmussen et al. 2011)https://paperpile.com/c/qxd37D/185J
Reich et al. 2011	Re11	106	16	(David Reich et al. 2011)https://paperpile.com/c/qxd37D/U3pk
Skoglund et al. 2014	S14	15	1	(Skoglund et al. 2014)
Xing et al. 2010	X10	92	4	(Xing et al. 2010)https://paperpile.com/c/qxd37D/oUpm
Xu et al. 2011	X11	28	3	(Xu et al. 2011)https://paperpile.com/c/qxd37D/L8Wc
Yunusbayev et al. 2012	Y12	183	14	(Yunusbayev et al. 2012)https://paperpile.com/c/qxd37D/VdBe

Yunusbayev et al. 2015	Y15	299	42	(Yunusbayev et al. 2015)https://paperpile.com/c/qxd37D/uc9O

Supplemental Table 1: Data Sources. Abbrev: Abbreviation; Ind: total number of individuals; Loc. Number of unique sample locations

Panel	Abb.	Ind.	Locations	SNPs	Grid Size (# of demes)	Resolution (km)	F_{ST}	Model-fit F_{ST} (adjacent demes)	Model-fit F_{ST} (500km)	Support (log-BF)
Afro-Eurasia	AEA	4697	370	19972	686	500	0.071	0.99%	0.99%	254,472
Central/Eastern Eurasia	CEA	2578	181	21045	1147	240	0.042	0.22%	0.42%	129,035
Western Eurasia	WEA	2049	122	26438	1437	120	0.010	0.75%	1.08%	46,210
South-East Asia	SEA	1054	58	7553	1388	120	0.037	0.29%	0.56%	13,654
Africa	AFR	749	71	20984	694	240	0.055	0.81%	1.18%	51,771
Southern Africa KhoeSan	SAKS	109	16	532343	227	120	0.025	0.32%	0.62%	2298
Southern Africa Bantu	SAB	30	11	65095	227	120	0.014	0.26%	0.56%	126

Supplemental Data Table 2: Analysis Panels. Abb. Panel Abbreviation. Res. Avg. distance between grid points (in km) ; Support: log Bayes factor in favor of complex vs constant migration model. Implied F_{ST} between adjacent demes based on posterior mean migration rates. Equation 19a from (Slatkin 1991) is used to calculate implied F_{ST} using a torus approximation: For F_{ST} (adjacent demes): $F_{ST}=(1+32m/S(d))^{-1}$ where S(d) is a function of the distance between demes and given by equation A12 in (Slatkin 1991). In the first column, we use S(1), in the second S(4) for highest and S(2) for medium resolution panels to get FST for demes at the lowest resolution (~500km).

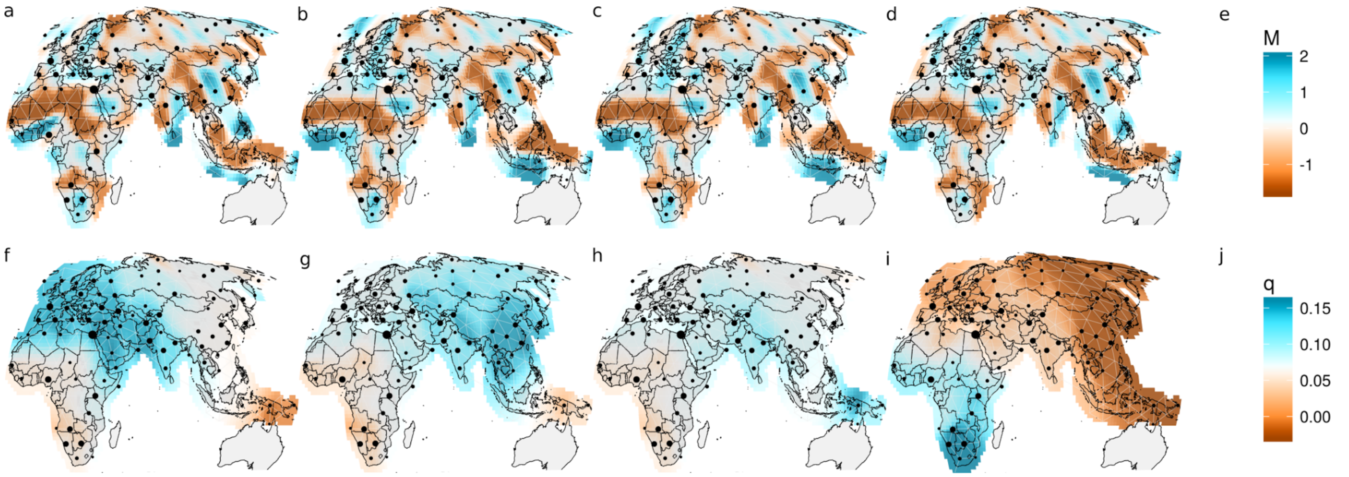

Supplemental Data Figure 1: Ascertainment bias. We run EEMS only using the Human Origin data (Lazaridis, Patterson, Mittnik, Renaud, Krause, et al. 2014), using SNPs ascertained in a French (a/f), Chinese (b/g), Papuan (c/h) and San(d/i) individual. Migration rate surfaces (a-d) remain robust, whereas the within-deme diversity surfaces (f-i) show highests diversity at the respective ascertainment location. e/j: scale bars for migration rates and within-deme diversity rate parameters, respectively.

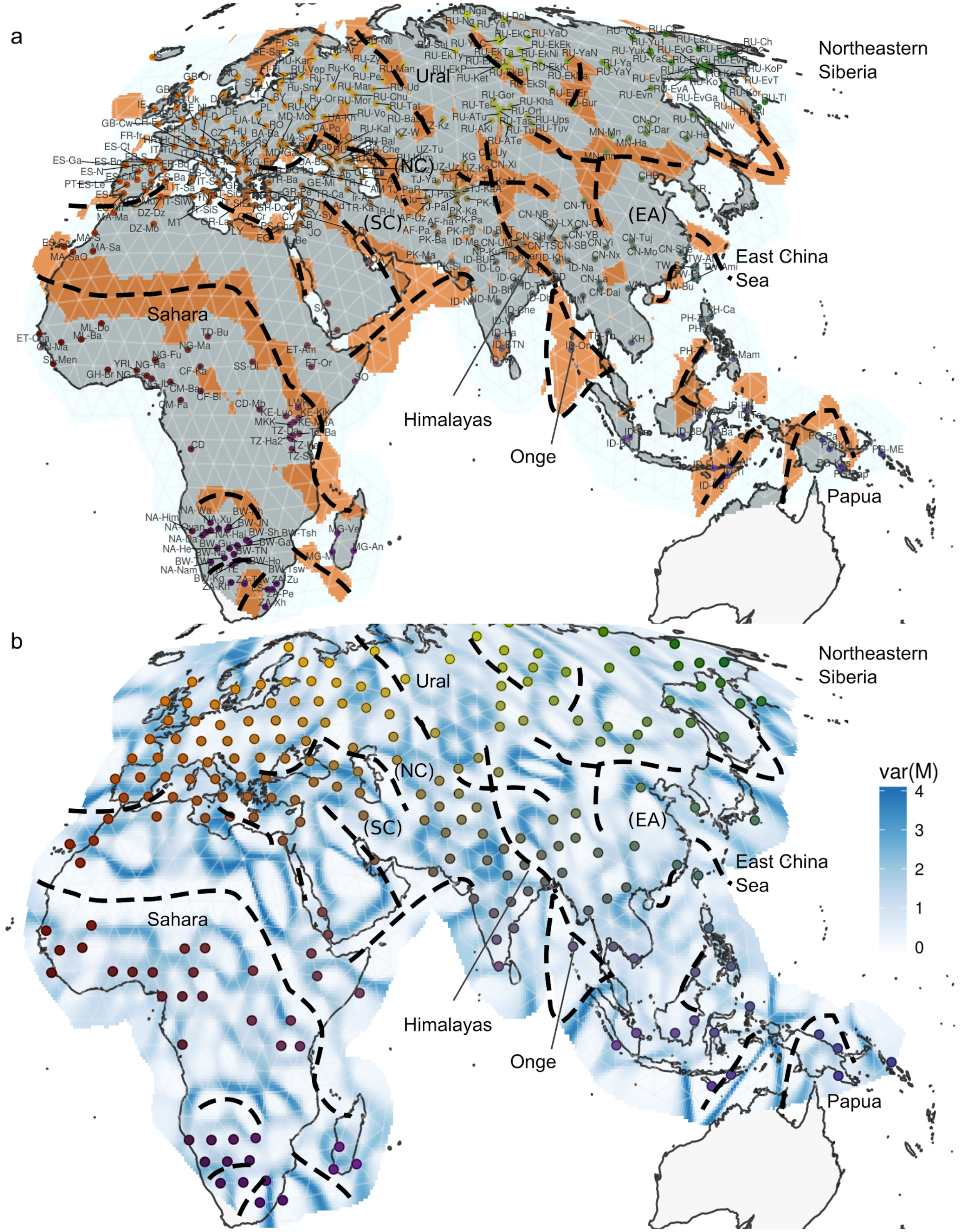

Supplemental Data Figure 2: a: Location of troughs (below average migration rate in more than 95% of MCMC iterations) are given in brown. Sample locations and EEMS grid are displayed. **b:** Posterior variance on migration rate parameters. Note that most significant features are in low variance regions, but that they are often surrounded by high-variance regions, implying the exact boundary of troughs is estimated with uncertainty. Grid-fitted sample locations are displayed. Annotation in both panels is identical to Figure 1a.

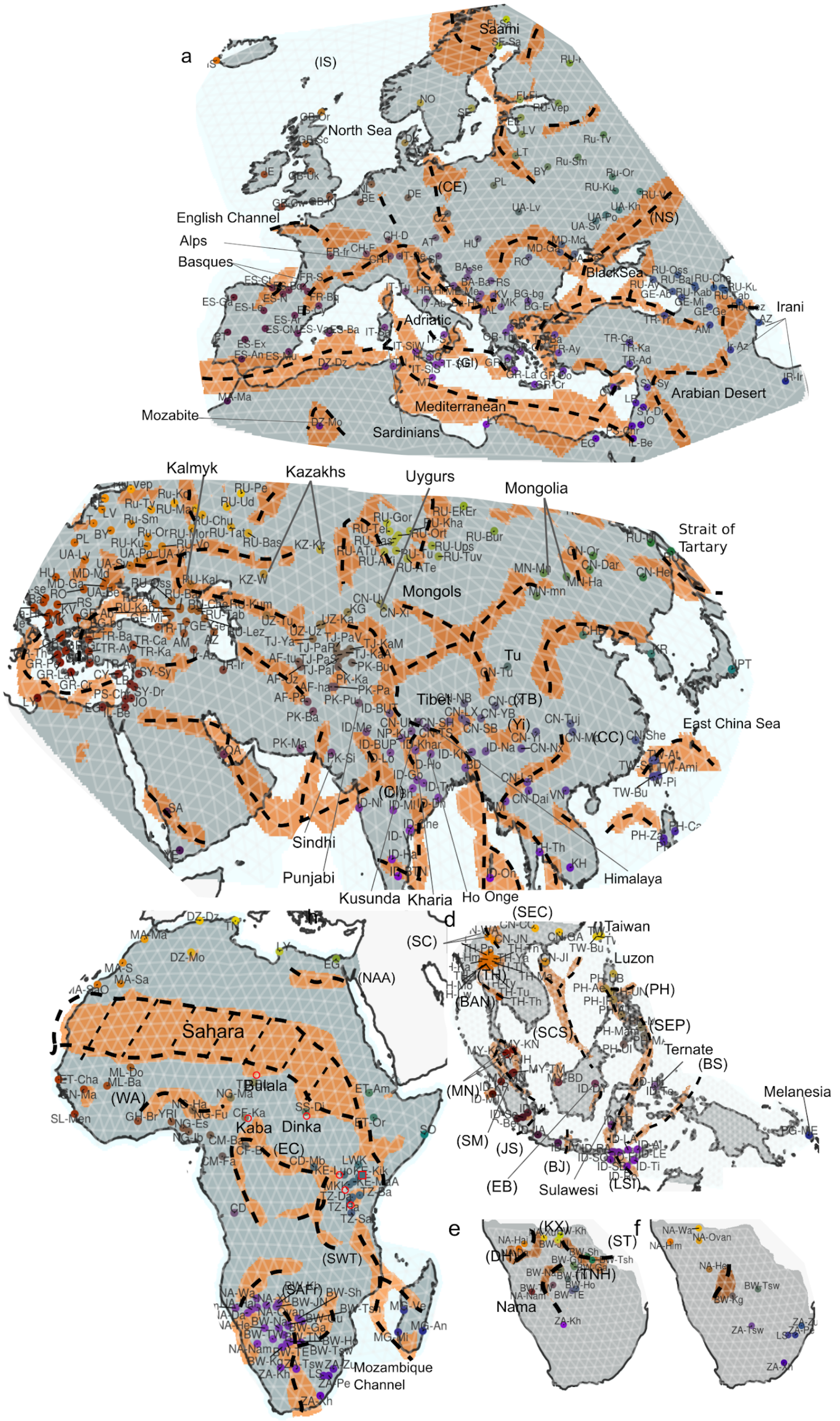

Supplemental Data Figure 3: Location of troughs (below average migration rate in more than 95% of MCMC iterations) are given in brown. Sample locations and EEMS grid are displayed for **a**: WEA **b**: CEA **c**: AFR **d:** SAHG and **e**: SEA analysis panels. Annotation in all panels is identical to Figure 2.

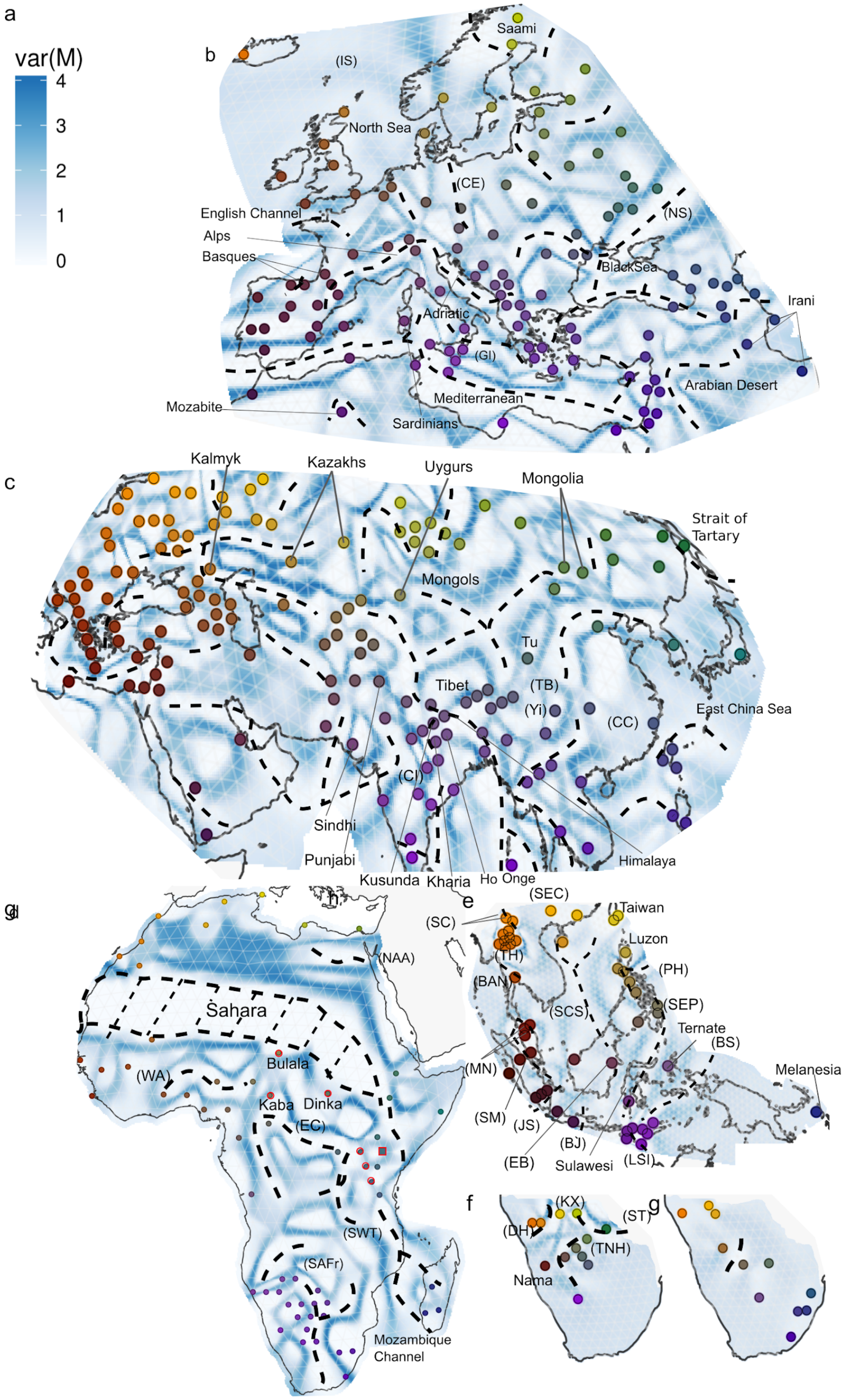

Supplemental Data Figure 4: Posterior variances in migration rate parameters. Grid-fitted sample locations are displayed .**a**: scale bar **b:** WEA **c**: CEA **d**: AFR **e:** SAHG and **f**: SEA analysis panels. Note that most significant features are in low variance regions, but that they are often surrounded by high-variance regions,

implying the exact boundary of troughs is estimated with uncertainty. Annotation of troughs and select features is identical to Figure 2.

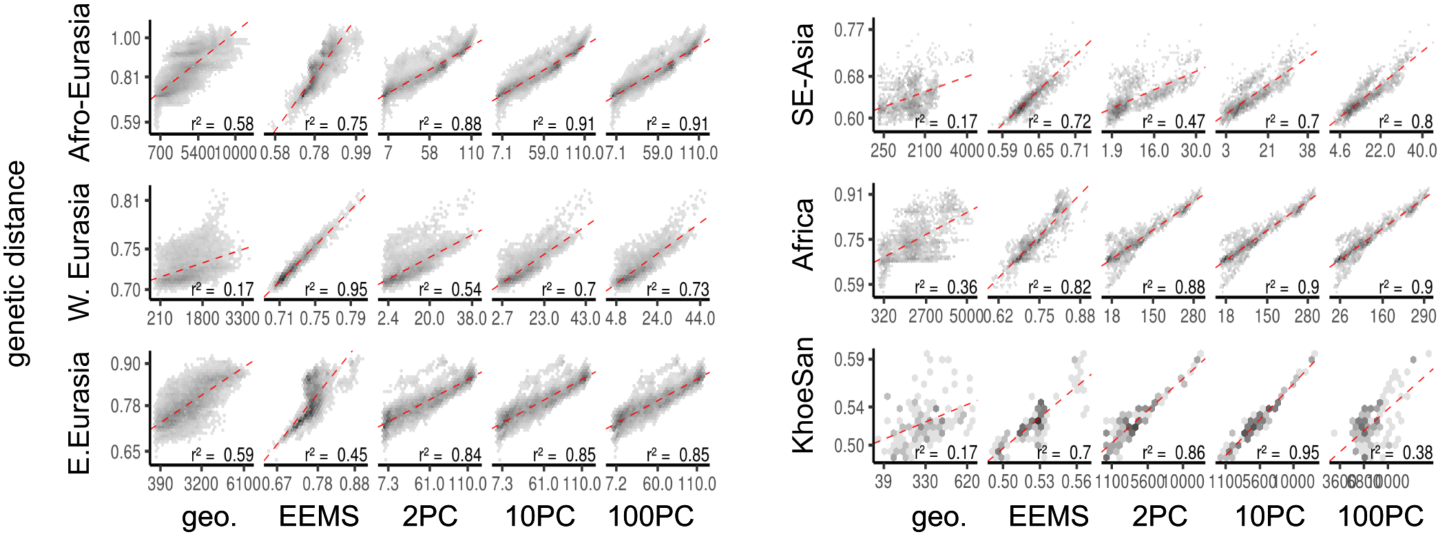

Supplemental Data Figure 5: Hex-binned scatterplots of genetic distance versus geographic distance (in km), predicted distance via EEMS model fit, and predicted distance via a ten-component PCA, for all panels. Darker areas correspond to bins with more points. The fit of a simple linear regression (red dashed lines) and r² are given.

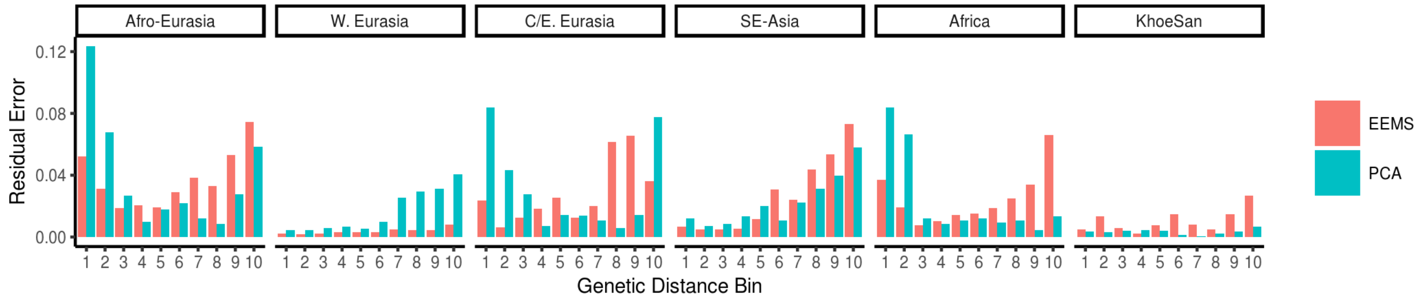

Supplemental Data Figure 6: Comparing Fit of PCA and EEMS. We show the relative error of EEMS (red) and PCA(blue, first 10 PCs) for all pairs, stratified by genetic distance. For each panel, all pairwise genetic distances were distributed in ten bins of equal size, for which we then computed the median absolute error of the fitted model vs the observed distances. For W. Eurasia and SE-Asia, EEMS fits uniformly better than PCA. In the Afro-Eurasian, Central/Eastern Eurasian and African panel, EEMS fitts better for smaller distances, but the fit is worse for larger distances. For the KhoeSan, EEMS fits worse than PCA for all distance bins.

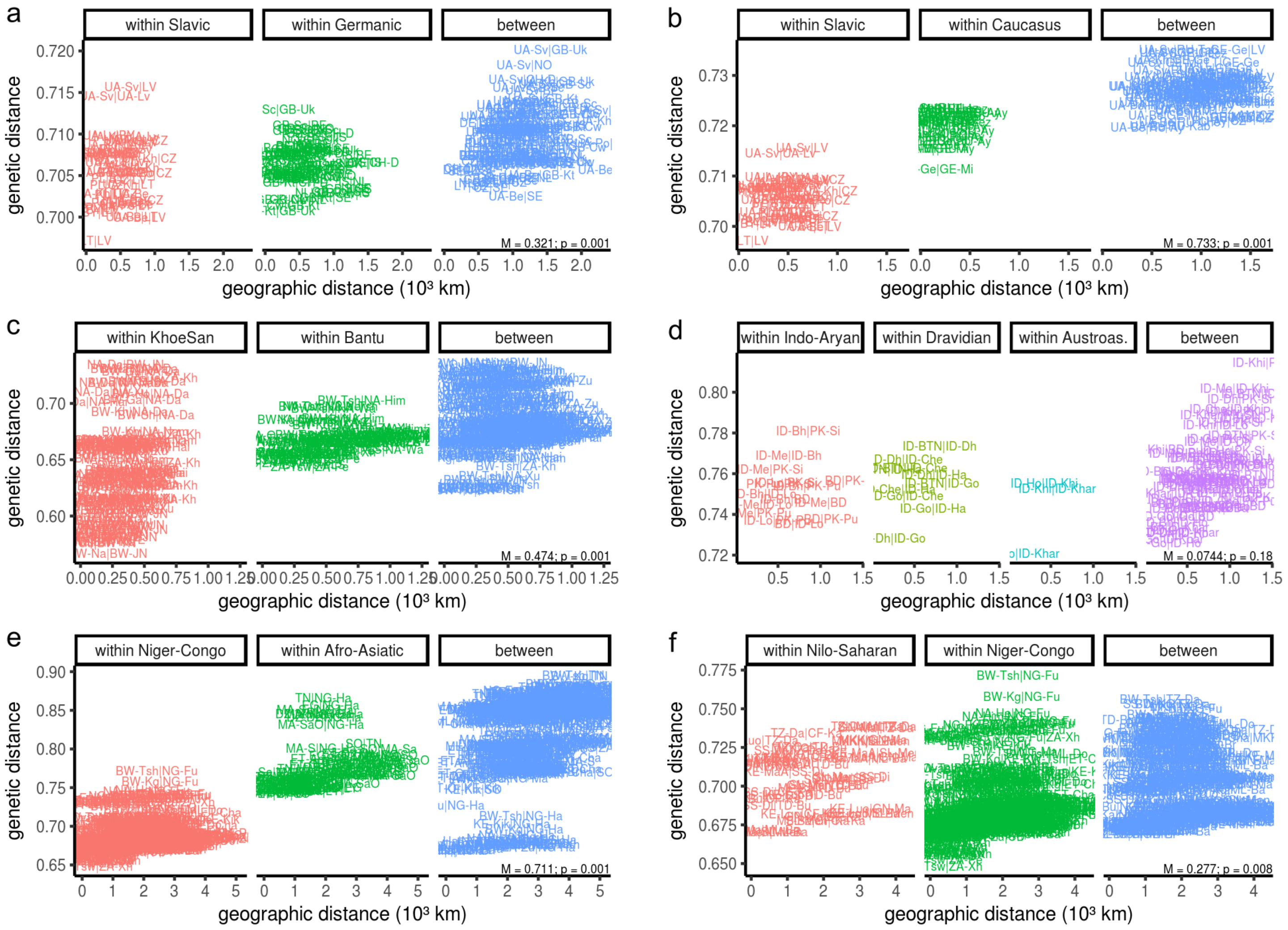

Supplemental Data Figure 7: Genetic vs. geographic distance within and between language groups. The eems-plots revealed several troughs aligning with differences in linguistic groups. We show the pairwise relationship of genetic and geographic differences within- and between adjacent language groups mentioned in the main text for a. Slavic and Germanic speakers (WEA panel) b. Slavic and Caucasus languages (WEA), c. KhoeSan and Bantu languages (Southern Africa) d. Indo-Aryan, Dravidian and Austroasiatic (CEA) e. Niger-

Congo and Afro-Asiatic (AFR) and f. Nilo-Saharan and Niger-Congo (AFR).

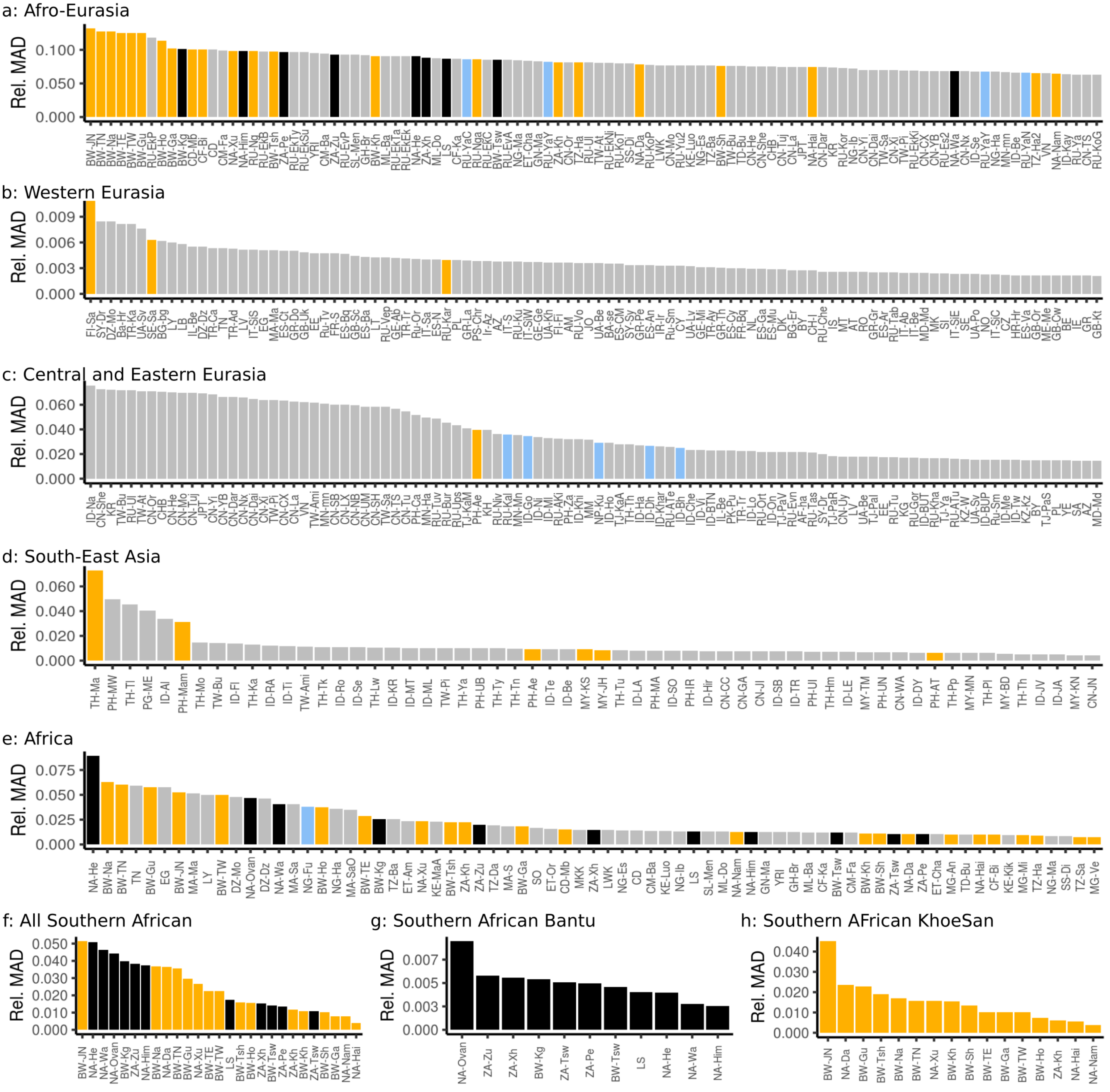

Supplemental Data Figure 8: EEMS-fit residuals. For each population, we show the median absolute deviation (MAD) of the observed vs EEMS-fitted genetic distances, normalized by the median distance for this population. yellow: Hunter-Gatherers; Black: Southern African Bantu speakers; Blue: Populations with a recent admixture or displacement.

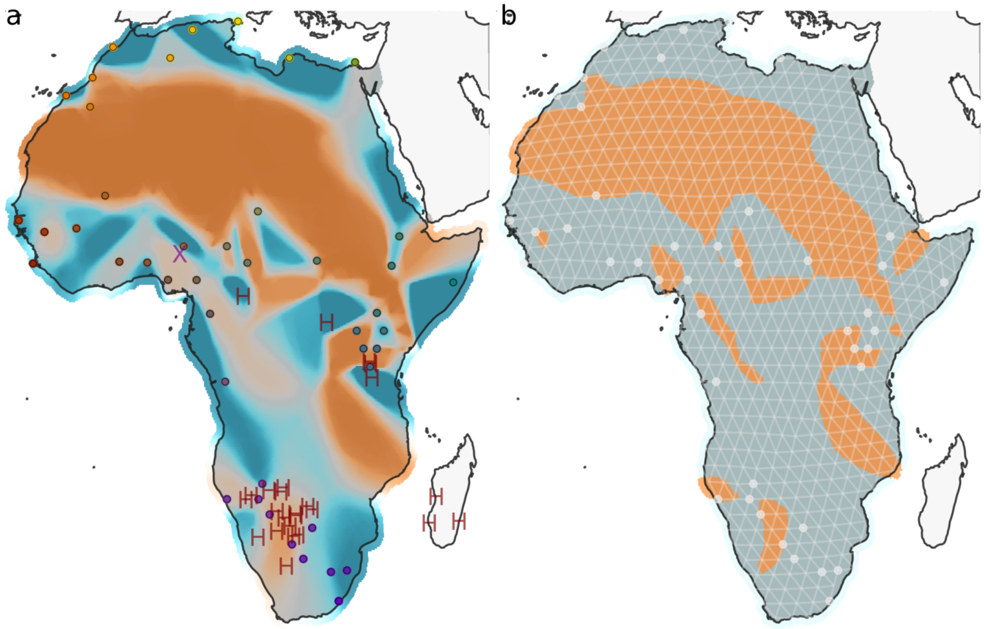

Supplemental Data Figure 9: Alternative Africa analysis. To assess the effect of populations that may not be modelled well by EEMS (admixed or hunter-gatherer populations), we provide supplemental analyses of Africa with several populations excluded from the model fit. **a:** EEMS-map and **b:** location of troughs for Africa. Excluded populations are annotated with H (Hunter-gatherers) and X (admixed). With this filtering (in particular removing the Hadza and Sandawe), the Eastern African trough between Afro-Asiatic speakers and Nilo-Saharan / Niger-Congo speakers (seen in Figures 1 and 2g) vanishes.
